# Exploring hazardous alcohol use and its determinants among health professionals in Bahir Dar, Northwest Ethiopia

**DOI:** 10.3389/fpsyt.2025.1511575

**Published:** 2025-02-20

**Authors:** Selam Koye, Techilo Tinsae, Melak Menberu, Minale Tareke

**Affiliations:** ^1^ Department of Psychiatry College of Medicine and Health Science, University of Gondar, Gondar, Ethiopia; ^2^ Department of Nursing, College of Medicine and Health Sciences, Bahir Dar University, Bahir Dar, Ethiopia; ^3^ College of Medicine and Health Sciences, Bahir Dar University, Bahir Dar, Ethiopia

**Keywords:** Hazardous alcohol use, health professionals, risk factors, psychological distress, khat use, Ethiopia

## Abstract

**Introduction:**

Alcohol consumption among health professionals can have serious and multifaceted impacts, affecting both the individual and their professional responsibilities. Despite its critical importance, there is limited research on the burden and contributing factors of hazardous alcohol use among health professionals in Ethiopia. This study aimed to evaluate the magnitude and determinants of hazardous alcohol use among health professionals in Bahir Dar City, Northwest Ethiopia.

**Method:**

An institution-based cross-sectional study was conducted from June 1-30, 2020, involving 384 health professionals. Participants were selected through a simple random sampling method, stratified by profession. Data were collected using a structured and semi-structured self-administered questionnaire, and hazardous alcohol use was assessed using the 10-item Alcohol Use Disorder Identification Test (AUDIT). Data entry was done using EpiData Version 4.6, and analysis was carried out with SPSS Version 23. Bivariable and multivariable logistic regression analyses were employed to estimate crude and adjusted odds ratios, respectively, with a 95% confidence interval. A p-value of less than 0.05 was considered statistically significant.

**Results:**

The burden of hazardous alcohol use among health professionals was 16.4% (95% CI: 13%-20.3%). Significant factors associated with hazardous alcohol use included having a family history of alcohol consumption (AOR=2.68, 95% CI: 1.27-5.66), mild psychological distress (AOR=2.7, 95% CI: 1.12-6.50), moderate/severe psychological distress (AOR=5.84, 95% CI: 2.08-16.37), lifetime Khat use (AOR=10.44, 95% CI: 3.96-27.53), and possessing a diploma-level education (AOR=3.56, 95% CI: 1.28-9.87).

**Conclusion:**

The high prevalence of hazardous alcohol use among health professionals in Bahir Dar City underscores an urgent need for targeted intervention and prevention strategies. Significant risk factors, including family history of alcohol use, psychological distress, Khat use, and educational level. To reduce hazardous alcohol use, the study recommends mental health programs, workplace policies promoting well-being, and integrated prevention and treatment strategies for alcohol and khat use.

## 1 Introduction

Hazardous alcohol use is defined as a pattern of alcohol consumption that increases the risk of harmful consequences for the user or others. It involves drinking at levels likely to cause physical, psychological, or social harm, even if these consequences have not yet occurred (1, 2). Hazardous alcohol use is associated with high mortality, morbidity, and physical injuries such as alcohol dependence, liver cirrhosis, cancers, accidents, and violence. Alcohol-related deaths exceed those caused by all other drugs combined globally. Additionally, alcohol use is a risk factor for various societal issues, including absenteeism from work, accidents, and loss of productivity (3, 4). Health professionals use various methods to aid patients, who are high-risk alcohol users. However, there is a positive association between health professionals’ alcohol-related health promotion activities and their personal attitudes towards alcohol, as well as their own alcohol use (5–7). Medical students often consume alcohol to cope with academic pressure, and some continue hazardous drinking when they become professionals (8–11). The impact of alcohol use among health professionals affects their relationships with coworkers, their work performance, and patient care. Furthermore, it influences the implementation of strategies for promoting health and preventing alcohol use in workplace relationships, cognition, and professionalism (4, 12, 13).

According to the World Health Organization’s (WHO) Report on Alcohol and Health, total alcohol consumption per capita rose rapidly from 5.5 Liters in 2005 to 6 Liters in 2016 globally. Alcohol use contributed to approximately 3 million deaths worldwide in 2016, and its impact on mortality surpasses that of tuberculosis, diabetes, hypertension, digestive diseases, road injuries, and violence. Mortality rates attributable to substances were highest for tobacco smoking, followed by alcohol and illicit drugs (14–17). The reasons for alcohol use among individuals include self-medication, negative affect, and work-related fatigue resulting from exposure to occupational stressors (18–20). Alcohol consumption in Ethiopia is comparable to global estimates and has shown a considerable increase in hazardous alcohol consumption (21). Ethiopians commonly consume traditional homemade alcoholic beverages such as Tella, Tej, Shameta, Bordie, Korefie, and Areki, in addition to known alcoholic beverages like wine and beer, particularly during traditional festivals such as Christian celebrations, weddings, birthdays, funerals, and for recreation (22).

The alcohol-attributable disease burden becomes even more significant when considering alcohol’s impact on the incidence and progression of HIV/AIDS, with alcohol responsible for 6% of all deaths and 5% of all disability-adjusted life years lost in the African Region. Alcohol’s role in HIV/AIDS-incidence highlights the need for strong policy responses to reduce the alcohol-related disease burden on the continent (23). The WHO report shows that large proportions of adults in most countries around the world consume alcohol. Consequently, around 5% of the world’s adult population suffers from alcohol use disorder, leading to an estimated loss of 257 years of healthy life per 100,000 population (14).

In the United States, health professionals, including physicians, face higher rates of alcohol abuse and dependency than the general population, driven by factors such as burnout, emotional exhaustion, peer pressure, long working hours, and low social support (24–26). In Belgium, medical specialists have high rates of hazardous alcohol use, including frequent binge drinking, exceeding consumption levels in the general population (27, 28). In Spain, hazardous alcohol use is more prevalent among male health professionals aged 56 and older and those with extensive work experience (29).

Similarly, Italian health professionals consume various types of alcoholic beverages hazardously, including wine, beer, and hard liquor. Some drink occasionally, while others consume alcohol regularly (30).

In Africa, studies from Nigeria and Kenya reveal that health professionals often consume alcohol at rates equal to or higher than the general population. In Nigeria, many medical specialists are hazardous drinkers, though most female professionals abstain. In Kenya, alcohol and tobacco are the most commonly used substances among health professionals, with some also using drugs like cannabis and sedatives (28, 31–33). In Ethiopia, a one study found that the prevalence of alcohol use disorders among health professionals was similar to that of the general population (21).

Despite the critical role of health professionals in addressing alcohol-related issues, their own alcohol consumption can impact their effectiveness. In Ethiopia, research on hazardous alcohol use among health professionals is scarce and often limited to severe alcohol use disorders or single tertiary hospitals. This study aimed to estimate the burden of hazardous alcohol use among health professionals in Bahir Dar City, Ethiopia, and examine its socio-demographic and occupational factors. The findings will support stakeholders in developing targeted alcohol prevention policies and provide baseline data for future research and program development.

## 2 Materials and methods

### 2.1 Study design and period

An institution-based cross-sectional Study Design was conducted from June 1 to 30, 2020.

### 2.2 Study area

The study was conducted in Bahir Dar City Administration, the capital of the Amhara Regional State, located in Northwest Ethiopia, approximately 565 km from Addis Ababa, the capital of Ethiopia. According to the 2007 Census conducted by the Central Statistical Agency of Ethiopia, the city has a total population of 221,991, of whom 81.16% are urban inhabitants, while the rest reside in the rural Kebeles surrounding Bahir Dar City (34). Previous studies indicate that the magnitude of problematic alcohol use in the city is high (35). Within Bahir Dar City, there are Felege Hiwot Comprehensive Specialized Referral Hospital (FHCSRH), Tibebe-Ghion Specialized Teaching Hospital (TGSTH), Addis Alem Primary Hospital (AAPH) and ten Health Centers. The City also has a total of 2,098 health professionals (HPs).

### 2.3 Population

#### 2.3.1 Source population

All health professionals actively engaged in health services within Bahir Dar City’s health institutions.

#### 2.3.2 Study population

All selected health professionals working in public health institutions in Bahir Dar City, including physicians, environmental health professionals, radiographers, laboratory technicians, health educators, nurses, psychiatrists, midwives, anesthetists, health officers, physiotherapists, and health educators working at Felege Hiwot Comprehensive Specialized Referral Hospital (FHCSRH), Tibebe-Ghion Specialized Teaching Hospital (TGSTH), Addis Alem Primary Hospital (AAPH), and the ten health centers in Bahir Dar City Administration.

### 2.4 Inclusion and exclusion criteria

#### 2.4.1 Inclusion criteria

All health professionals in the study setting who were present during the data collection period.

#### 2.4.2 Exclusion criteria

Health professionals who were on various types of leave or attending training during the data collection period were excluded.

### 2.5 Sample size determination

Both the associated factors and the burden of hazardous alcohol consumption were based on a previous study conducted on health professionals in Ethiopia (32). The calculated sample size using associated factors for the burden of hazardous alcohol use was greater than the sample size determined using the Single-Population-Proportion-Formula. Therefore, the final sample size for this study was 396 health professionals, including a 10% non-response rate (**Table 1**).

**Table 1:** Sample size for factors associated with hazardous alcohol use among health professionals in Bahir Dar City, Northwest Ethiopia, 2020.

No.	Significant variable	Assumptions	Total sample size
1	Using single-population-proportion-formula	▪ Burden of alcohol use disorder among health professionals (32) = 8% ▪ Z= 1.96 ▪ Margin of error= 5% ▪ CI= 95%	113
1	Current kahat chewing (chewer = exposed; (Not chewer = unexposed)	▪ Two-sided confidence level = 95% ▪ Power = 80% ▪ % Outcome in unexposed = 2.9 ▪ % Outcome in exposed = 22.5 ▪ **Odds ratio = 3.9**	108
2	Psychological Distress (Distressed = exposed; (Not distressed = unexposed)	▪ Two-sided confidence level = 95% ▪ Power = 80% ▪ % in outcome in unexposed = 2.6 ▪ % outcome in exposed = 16.7 ▪ **Odds ratio = 7.5**	164
3	Professional interest (Interested = exposed; (Not interested = unexposed)	▪ Two-sided confidence level = 95% ▪ Power = 80% ▪ % Outcome in unexposed = 4.0 ▪ % Outcome in exposed = 16.7 ▪ **Odds ratio = 0.29**	360
4	Current Tobacco Use (User = exposed; (Not user = unexposed)	▪ Two-sided confidence level = 95% ▪ Power = 80% ▪ % Outcome in unexposed = 5.4 ▪ % Outcome in exposed = 32.4 ▪ **Odds ratio = 8.4**	78

### 2.6 Sampling technique and procedure

All health professionals in the three hospitals and ten health centers were stratified based on their working departments. A stratified sampling technique was used to select the study units in each stratum. Based on the number of departments in each Stratum, a proportional allocation of the total sample size was conducted to achieve the required sample size. Finally, the determined sample size for each Stratum was selected using a simple random sampling technique.

### 2.7 Variables (dependent and independent variables)

#### 2.7.1 Conceptual framework

**Figure 1:**
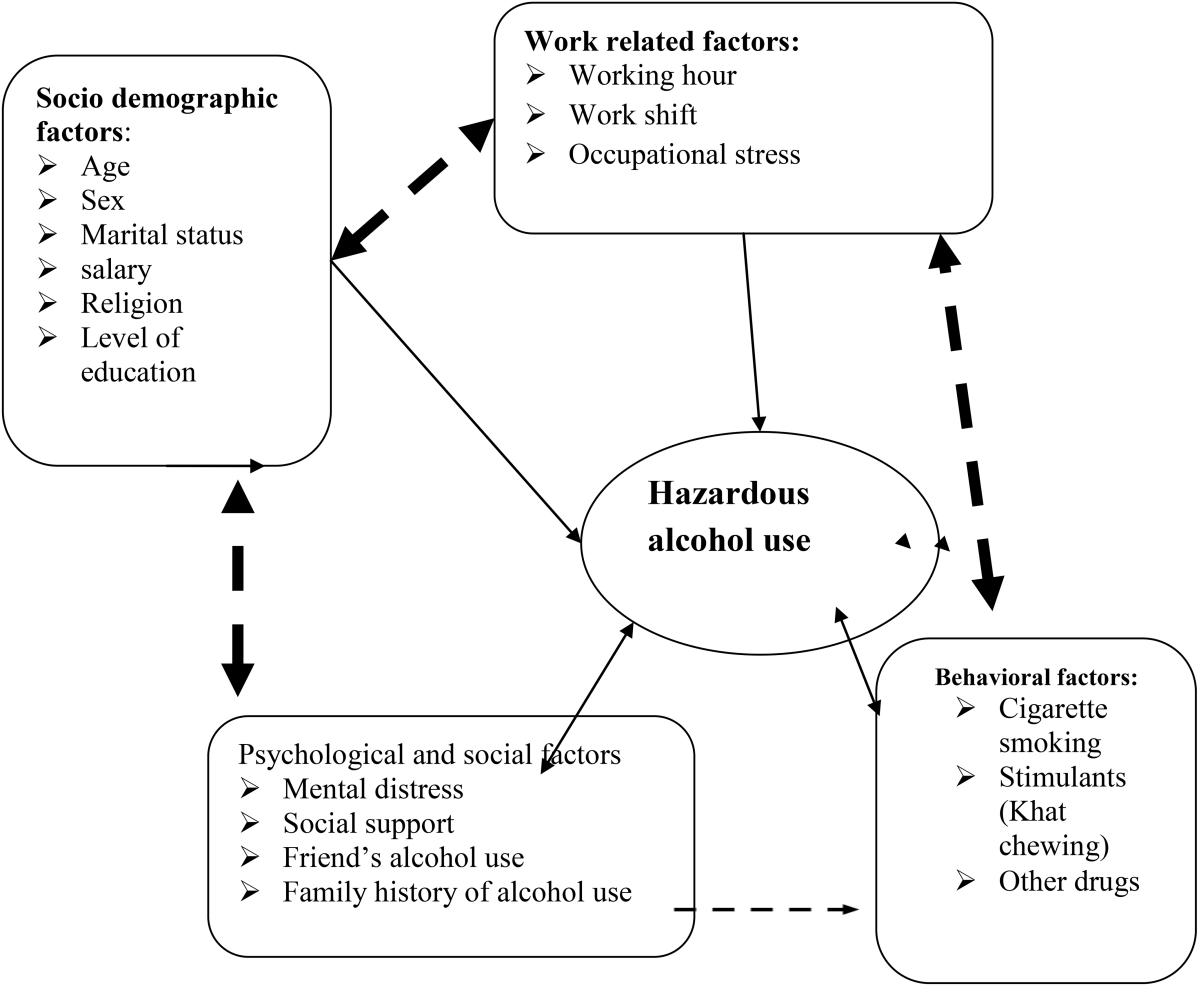
Conceptual framework for hazardous alcohol use and associated factors among health professionals in Bahir Dar City, 2020 (36–38).

Studies indicate that factors associated with health professional’s alcohol consumption are work related factors, socio demographic, behavioral and mental health conditions (**Figure 1**).

### 2.8 Data collection instrument

The Alcohol Use Disorders Identification Test (AUDIT) is a 10-item alcohol screening tool developed by the WHO, which focuses on identifying alcohol use disorders within the last 12 months (39). It is highly effective in detecting less severe drinking problems, such as hazardous drinking, harmful drinking, and alcohol dependence (sensitivity: 94.1%; specificity: 91.7%). AUDIT is crucial for identifying problematic alcohol use at an early stage, allowing professionals to take preventive measures and reduce the associated problems. The AUDIT consists of three parts: the first three questions (1–3) assess the quantity and frequency of alcohol consumption (Hazardous Alcohol Use), the next three questions (4–6) assess signs of alcohol dependence, and the final four questions (7–10) assess alcohol-related problems (Harmful Alcohol Use). Each question offers a response category ranging from 0 to 4, with the first response scoring 0 (never), the second scoring 1 (less than monthly), the third scoring 2 (monthly), the fourth scoring 3 (weekly), and the last response scoring 4 (daily or almost daily). For questions 9 and 10, which have only three responses, the scoring is 0, 2, and 4.

The AUDIT was adapted to fit the country-specific context, as recommended by the WHO. Traditional Ethiopian beverages were converted to equivalent alcohol units. To determine the number of standard drinks consumed by the health professionals, question 2 was modified to include “Type of alcohol” and “Its amount.” The response was converted into standard drinks, where one standard drink equals: ‘Tella’ (1 glass of ‘borde,’ ‘cheka,’ ‘korefe,’ ‘filter,’ or ‘tsewa’), ‘Tej’ (1/2 ‘Berele’), ‘Areke’ (1 ‘melkiya’), regular beer (330 ml or 1 bottle), draft beer (1 single), spirits (30 ml of whisky, gin, uzo, vodka, etc.), or wine (120 ml). A standard drink is defined as containing approximately 10g of ethanol.

Following the recommended scoring, a total AUDIT-score of eight or more was used to define probable alcohol use disorder (40). Studies have shown that a cut-off score of 8 has favorable Sensitivity and acceptable Specificity for current ICD-10 alcohol use disorders and future harm. In Kenya, a cut-off score of 8 was used to identify hazardous alcohol use in the general population (36), while in Nigeria, a cut-off score of 5 or above was used for health workers (29). AUDIT is widely used in low- and middle-income-Countries, and nonstandard cut-off scores may be appropriate in these settings (37). This study used a Cut-off of 8 and above to identify hazardous alcohol users, with an internal consistency (Cronbach’s alpha) of 0.76.

Psychological distress was assessed using the Kessler-10 (K10), which measures global distress based on questions about anxiety and depressive symptoms experienced in the most recent 4-week period (Cronbach’s alpha) of 0.92. This tool has been validated and used by other researchers among rural populations in Ethiopia (38).

Health professionals’ occupational stress was assessed using the Perceived Stress Scale (PSS), developed by the WHO, to examine personal stress levels. The 10-item PSS was used to assess current occupational stress. To focus on work-related stress, the questions were modified to reflect stress in the workplace rather than at home. The PSS-10 scores are obtained by reversing the scores of the four positive items (questions 4, 5, 7, and 8) and summing across all 10 items. Scores around 13 are considered average, while scores of 20 or higher indicate high stress (Cronbach’s alpha) of 0.78. This tool has also been used by other researchers to measure health professionals’ Stress levels in Ethiopia (39, 40).

Social support among health professionals was assessed using the Oslo Social Support Scale (OSS-3), a brief measure consisting of three items (41). Additional factors, such as socio-demographic and economic characteristics, were measured using structured questionnaires, developed based on various literature sources.

### 2.9 Data-collection-procedure

The data in this study are organized into two main categories: dependent variables and independent variables. The dependent variable (hazardous alcohol consumption) is influenced by a range of independent variables, categorized into Sociodemographic, work-related, psychological and social, and behavioral factors. This approach helps to understand the multifaceted nature of alcohol consumption and the complex interplay of different risk factors. The data was collected using a self-administered structured questionnaire. Five BSc psychiatric nurses participated as data collectors, and one supervisor oversaw the supervision. Two days of training were provided for the data collectors and the supervisor. During the training, the study’s objective was discussed, along with the data collection methods, tools, and procedures for handling ethical issues. Each question in the structured questionnaire was reviewed in detail, and any doubts were clarified. The responsibilities of the supervisor were also thoroughly explained.

### 2.10 Data quality control

A Pre-test was conducted with 5% (19) of the sample size at Adet Primary Hospital, which is outside the study area, to identify potential issues with the data collection tools. Any necessary amendments to the questionnaire were made based on the Pre-test-results, and the questionnaires used in the pre-test were not included in the main study analysis. Both the supervisor and principal investigator regularly supported and monitored the data collectors, ensuring that each completed questionnaire was checked, and feedback was provided to the data collectors the following morning. The collected data was properly handled, reviewed, and checked for completeness and consistency by the supervisor and principal investigator each day. Incomplete data was excluded from the study. The purpose and importance of the study were explained to the participants. All research instruments, initially in English, were translated into Amharic and then back-translated into English by language experts to maintain consistency.

### 2.11 Data processing, analysis and presentation

The collected data were checked for completeness and consistency. The data were edited, cleaned, coded, and entered into EpiData version 4.6, then exported to SPSS version 23 for analysis. The mean, frequencies, and percentages of the variables were estimated using descriptive statistical analysis. All explanatory variables with a p-value less than 0.2 in the bivariate logistic regression analysis were included in the multivariate logistic regression model. This was done to account for and control the potential influence of confounding factors. The strength of the associations was measured by odds ratios with 95% confidence intervals, and a p-value of less than 0.05 was considered statistically significant. Results were presented in the form of tables, figures, and charts, using frequency and summary statistics such as means and percentages to describe the study population in relation to relevant variables, which were discussed in the context of previous findings.

### 2.12 Ethical consideration

Ethical approval was obtained from the Ethical Review Board of Bahir Dar University College of Medicine and Health Sciences, and a permission letter was received from the Amhara Regional Bureau of Health. Written informed consent was obtained from the study participants, based on their voluntariness, and they had the right to discontinue participation at any time. To ensure strict confidentiality, participants were not required to provide their names. The data provided by the participants was used solely for research purposes.

## 3 Result

### 3.1 Socio-demographic and economic characteristics of study participants

Out of 396 sampled health professionals, 384 completed the questionnaire, resulting in a response rate of 97%. Among the total participants, 62.2% were male. Of the participants, 56.5% were aged between 26 and 30 years, with a mean age of 29 years (SD ±4.98). Approximately 227 participants (59.1%) were single, and 319 (83.1%) held a Bachelor’s degree, with an average monthly income of 6,071 ETB (SD ±2,311). Among the total sample, 43% were nurses, and 16.4% were physicians (**Table 2**).

**Table 2:** Socio-demographic and economic characteristics of health professionals in Bahir Dar City, Northwest Ethiopia, 2020 (n=384).

Variable		Number	Percent
Sex	Male	239	62.2
	Female	145	37.8
Age	≤25	81	21.1
	26-30	217	56.5
	31-35	53	13.8
	≥36	33	8.7
Marital status	Single/divorced/widowed/separated	227	59.1
	Married	157	40.9
Religion	Orthodox	356	92.7
	Muslim	22	5.7
	Others	6	1.6
Ethnicity	Amara	360	93.8
	Others	24	6.3
Profession	Physician	63	16.4
	Nurse	165	43.0
	Midwifery	38	9.9
	Anesthetic	11	2.9
	Health Officer	16	4.2
	Pharmacy	26	6.8
	Laboratory	27	7.0
	Others	38	9.9
Educational Status	Diploma	29	7.6
	1^st^ Degree	319	83.1
	2^nd^ Degree and above	36	9.4
Income	<5000ETB	140	36.5
	5000-10,000ETB	217	56.5
	>10,000ETB	27	7.0
Currently living with	Alone	195	50.8
	Family	173	45.1
	Friends	16	4.2
Family member alcohol drink	Yes	63	16.4
	No	321	83.6
Friend alcohol drink	Yes	92	24.0
	No	292	76.0

### 3.2 Psychological, behavioral and work related characteristics of health professionals

About 66.4% of Health-professionals work more than 50 hours per week, and approximately 50% work on weekends for two to three days each month. More than half (62.8%) of the health professionals reported experiencing Stress at work. Among the participants, 17 (4.4%) were current tobacco users, and 19 (4.9%) were khat users. Additionally, 21% of the total sample reported psychological distress (**Table 3**).

**Table 3:** Psychological, behavioral, and work-related characteristics of health professionals in Bahir Dar City, Northwest Ethiopia, 2020 (n=384).

Psychological, behavioral and work-related variables		Frequency	Percent
Work experience	1-7 Years	283	73.7
	8-14 Years	86	22.4
	≥15 Years	15	3.9
Workload	39Hr/week	84	21.9
	40-50Hr/week	45	11.7
	>50Hr/week	255	66.4
Night-shift	No night duty	30	7.8
	one to three days/week	168	43.8
	4 or more days/week	186	48.4
Weekend-duty	0-1 days per a month	65	16.9
	2-3 days/month	192	50.0
	>=4 days/month	127	33.1
Occupational Stress	Yes	241	62.8
	No	143	37.2
Psychological Distress	No	304	79.2
	Mild	51	13.3
	Moderate and severe	29	7.6
Current Tobacco use	Yes	17	4.4
	No	367	95.6
Current Khat use	Yes	19	4.9
	No	365	95.1`
Lifetime Tobacco use	Yes	27	7.0
	No	357	93
Lifetime Khat use	Yes	32	8.3
	No	352	91.7

### 3.3 Burden of hazardous alcohol use

The burden of hazardous alcohol use in the current study was 16.4% (95% CI: 13.0%, 20.3%). Among the hazardous alcohol users, about 76.2% were male. Among the participants 103 participants (27.6%) were Abstainers and 215 (56.0%) were Social Drinkers.

### 3.4 Type of alcohol and reasons for alcohol drink given by health professionals

**Figure 2:**
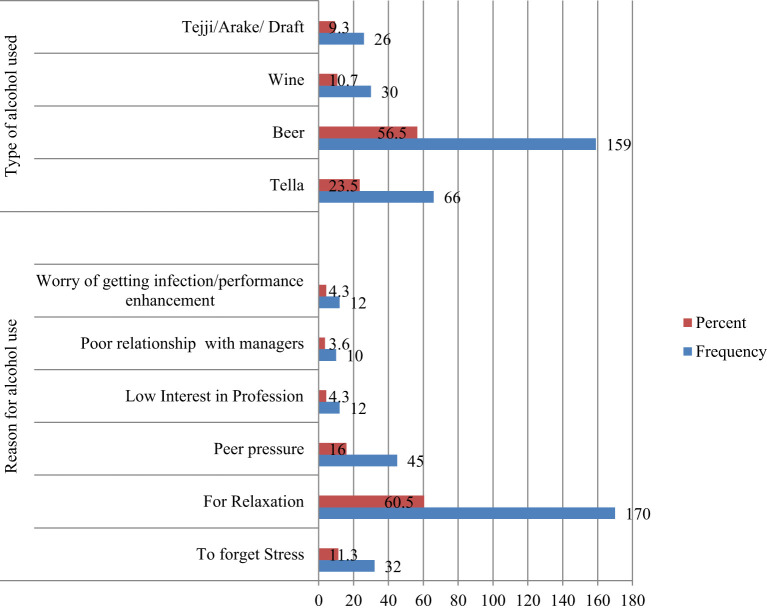
Reasons provided by health professionals for using alcohol and Types of alcohol used in the last 12 months, Bahir Dar City, Northwest Ethiopia, 2020 (n=281).

The most commonly used types of alcohol were beer (41.4%), followed by Tella (a homemade Ethiopian alcoholic drink) at 17.2%. The most commonly reported reason for alcohol consumption among participants was for relaxation (60.5%) followed by peer pressure (16%) (**Figure 2**).

### 3.5 Factors associated with hazardous alcohol use

Bi-variable logistic regression analysis showed that sex, marital status, current living status, educational status, psychological distress, family alcohol use, friend alcohol use, lifetime khat use, social support, and occupational stress were associated with hazardous alcohol use, with a p-value of <0.2. In the multi-variable logistic regression, educational status, psychological distress, family alcohol use, and lifetime khat use were significantly associated with hazardous alcohol use, with a p-value of <0.05.

In this study, participants with mild psychological distress were three times more likely (AOR 3.05, 95% CI: 1.35, 6.92) to have hazardous alcohol use compared to those with no psychological distress. Participants with moderate or severe psychological distress were seven times more likely (AOR 6.9, 95% CI: 2.7, 17.57) to have hazardous alcohol use than those with no psychological distress.

Regarding family alcohol-use: Respondents with a family history of alcohol use were about three times more likely (AOR 2.54, 95% CI: 1.22, 5.3) to have hazardous alcohol use than those without a family history of alcohol use.

Concerning the educational status of the participants, those with a diploma were four times more likely (AOR 3.56, 95% CI: 1.28, 9.87) to use alcohol hazardously compared to those with a Bachelor’s degree or higher. Respondents who had ever chewed khat were twelve times more at risk of being hazardous alcohol users than those who had never used khat in their lifetime (AOR 11.87, 95% CI: 4.7, 29.96) (**Table 4**).

**Table 4:** Bi-variable and multi-variable logistic regression: Factors associated with hazardous alcohol use among health professionals working in Bahir Dar, Northwest Ethiopia, 2020 (n=384).

Selected Variables		Hazardous alcohol use			
		YES	NO	COR (95%CI)	AOR (95% CI)
		N (%)	N (%)		
Sex	Male	48 (76.2)	191 (59.5)	2.178 (1.17, 4.05)	0.722 (0.3,1.68)
	Female	15 (23.8)	130 (40.5)		1
Psychological distress	No	33 (52.4)	271 (84.4)	1	1
	Mild	14 (22.2)	37 (11.5)	3.11 (1.52,6.34)	3.27 (1.38,7.77)*
	Moderate and sever	16 (25.4)	13 (4)	10.11 (4.47,22.86)	6.0 (2.31,15.55)*
Family alcohol use	Yes	23 (36.5)	40 (63.5)	4.04 (2.19,7.44)	2.68 (1.27-5.66)*
	NO	40 (12.5)	281 (87.5)	1	1
Friend alcohol use	Yes	32 (50.8)	60 (18.7)	4.5 (2.5,7.9)	1.76 (0.85,3.65)
	No	31 (49.2)	261 (81.3)	1	1
Educational status	Diploma	9	20	2.51 (1.09,5.8)	3.56 (1.28, 9.87)*
	First degree and above	54	301		
Life time chat use	YES	20 (31.7)	12 (3.73)	11.97 (5.47,26.22)	10.44 (3.96,27.5)*
	NO	43 (68.3)	309 (96.3)	1	1
Social support	POOR	30 (47.6)	71 (22)	3.63 (1.74,7.56)	2.06 (0.86,4.95)
	Moderate	21 (33.3)	147 (46)	1.23 (0.58,2.60)	0.90 (0.37,2.21)
	Strong	12 (19.04)	103 (32)		1
Occupational stress	YES	52 (82.5)	189 (58.9)	3.30 (1.66,6.565)	1.21 (0.54,2.88)
	NO	11 (17.5)	132 (41.1)		1
Marital status	Married	21 (33.3)	136 (42.3)		1
	Single/divorced/widowed/separated	42 (67.7)	185 (57.6)	1.47 (0.83,2.59)	0.74 (0.36,1.52)
Living with	Alone	42 (66.7)	153 (47.7)	2.19 (1.24,3.88)	1.97 (0.73,5.28)
	Family/Friend	21 (33.3)	168 (52.3)		1

*Bold values = P<0.05.

## 4 Discussion

A study conducted among health professionals in Bahir Dar, Northwest Ethiopia, found a 16.4% prevalence of hazardous alcohol use (95% CI: 13%-20.3%), indicating a significant public health concern. Among the participants, 76.2% of those with hazardous alcohol use were male. The possible justification for this could be related to gender differences in drinking patterns, where males are often more likely to engage in risky drinking behaviors due to social, cultural, and biological factors. In Ethiopia particularly in Amhara region societies, alcohol consumption is more socially accepted and normalized among men, and they may be exposed to more opportunities for drinking. Additionally, males may experience different stressors or coping mechanisms, such as greater social pressure or higher rates of alcohol use in social settings, which could contribute to the higher prevalence of hazardous alcohol use in male than women. Key factors associated with increased risk included psychological distress, family history of alcohol use, educational status, and a history of khat use. These findings underscore the need for targeted interventions addressing mental health, familial and cultural influences, and the risks of concurrent substance use within this population.

The burden identified in this study aligns with findings from various studies. For instance, a study conducted among Belgian medical specialists reported a hazardous alcohol use rate of 18% (27), while Danish physicians and American surgeons reported rates of 19% (40) and 19.55% (42), respectively. Additionally, a study in Ambo town, Ethiopia, found a burden of 13.1% (43). A study among Belgian medical specialists reported an 18% rate of hazardous alcohol use, aligning with the 16.4% rate in Bahir Dar. This similarity suggests that health professionals globally may face common stressors—long hours, high responsibility, and emotional demands—that lead to alcohol use as a coping mechanism. Additionally, the social acceptance and availability of alcohol in both Belgium and Ethiopia may contribute to these comparable rates. In Denmark, physicians reported a 19% rate of hazardous alcohol use, slightly higher than the 16.4% in Bahir Dar. This highlights the global impact of occupational stress and burnout in the medical profession, leading to alcohol use as a coping mechanism. Denmark’s strong drinking culture, where alcohol is socially accepted and normalized, parallels Ethiopia’s societal acceptance of alcohol, possibly explaining the similar rates despite geographical differences. Studies in various settings highlight the global nature of occupational stress and its link to hazardous alcohol use among health professionals. American surgeons reported 19.55% prevalence, closely aligning with Bahir Dar’s 16.4%, reflecting the intense pressure, long hours, and high-stakes decision-making shared across healthcare settings. Similarly, Ethiopian studies show regional variations, with Ambo town reporting a slightly lower rate of 13.1%. This difference may be due to Bahir Dar’s greater alcohol availability, stronger social drinking culture, and the influence of khat use, which is associated with increased alcohol consumption. Despite geographical and cultural differences, the findings underscore that professional stressors universally drive hazardous drinking, influenced further by cultural norms and substance availability.

The current study’s prevalence of hazardous alcohol use (16.4%) is higher than rates reported in Ethiopia’s general population (10%) (21), and among health professionals at Jimma tertiary hospital (8.1%) (32). Differences may be due to methodology and measurement tools; the Jimma study used the CAGE questionnaire, which detects more severe drinking issues, while this study employed AUDIT. Additionally, disparities with a Brazilian study (10.6%) (44) could stem from sample size differences, as the Brazilian study’s larger sample may better represent its population. Variations in study design, such as systematic review versus cross-sectional methods, also likely contribute to these differences. This study’s findings (16.4%) are lower than a community-level Ethiopian study reporting 21% (42). The difference may stem from variations in educational levels, with health professionals potentially having greater awareness of alcohol-related risks. Additionally, the use of different measurement tools (FAST vs. AUDIT) and the rural context of the community study, a known risk factor for alcohol consumption, likely contribute to this discrepancy. This study’s findings (16.4%) are lower than those among Spanish healthcare providers (27.8%) (15) and the Australian physician (26.7%) (2). The higher rates in Spain may reflect a prevalence exceeding that of the general population and the study’s larger sample size (1760 participants). Differences with Austria could be due to varying AUDIT cut-off points and greater alcohol accessibility, which is linked to higher consumption rates.

Psychological distress is a significant predictor of hazardous alcohol use, as health professionals under high stress may turn to alcohol as a maladaptive coping mechanism (8, 28, 32, 45). Demanding work environments, long hours, trauma exposure, and decision-making pressures contribute to burnout and emotional discomfort, leading some to self-medicate with alcohol for its temporary sedative effects. Stigma around seeking mental health support and limited access to such resources often push professionals to manage distress privately with alcohol. In culturally permissive settings like Ethiopia, alcohol’s social acceptance further normalizes its use. A lack of stress management programs and healthy coping strategies exacerbates the issue, despite their medical knowledge. Family alcohol use is strongly associated with hazardous drinking, as individuals often learn unhealthy behaviors through social learning, observing family members who drink (5, 46). Health professionals from such backgrounds may view alcohol use as normal or a coping mechanism for stress. This early exposure can lead to similar patterns later, especially under professional stress. A family history of alcohol use may also suggest a genetic predisposition, increasing the likelihood of hazardous drinking. Additionally, these individuals may lack healthier coping strategies, further increasing their risk of turning to alcohol.

Educational status is a key factor in hazardous alcohol use, with lower education levels linked to less awareness of alcohol’s risks (32, 47). Diploma-level health professionals may be at higher risk due to factors like physically demanding work, long hours, job insecurity, and fewer career advancement opportunities. These stressors, combined with lower earnings and limited access to support systems, may drive them to use alcohol as a coping mechanism. Additionally, they may have received less education on alcohol’s risks and could experience isolation, leading them to use alcohol to socialize or escape stress. Khat chewing was strongly linked to hazardous alcohol use, with individuals who chewed khat being twelve times more likely to engage in risky drinking. This association may stem from khat use during student years, later replaced by alcohol due to professional demands or social norms. Khat, often consumed with alcohol, may encourage health professionals to drink to enhance or balance its stimulating effects (48). In cultures where both behaviors are common, alcohol use becomes normalized, increasing the likelihood of hazardous consumption. Additionally, khat’s stimulant effects can lead to anxiety or crashes, prompting individuals to self-medicate with alcohol. Social settings where khat and alcohol use overlap further increase the risk, making it a coping strategy for stress and emotional distress.

To address hazardous alcohol use among health professionals in Bahir Dar and other regions of Ethiopia, targeted, health system-specific strategies are essential. These should include: Establish mental health programs, train counselors for healthcare workers, and implement regular well-being assessments to support at-risk individuals. Introduce workplace policies restricting alcohol at work events, create confidential reporting systems, and ensure access to rehabilitation programs. Promote work-life balance through regulated working hours and leave policies. Develop integrated prevention and treatment programs, focusing on both alcohol and Khat use, with combined behavioral therapy. Conduct studies comparing alcohol use patterns in Ethiopia with other countries, focusing on work-related stress, cultural norms, and mental health service availability, and adapt international best practices to Ethiopia’s context.

### 4.1 Limitation of the study

The major limitation of this study was that occupational stress was assessed using the Perceived Stress Scale, even though it was adapted for workplace stress. Another limitation may be social desirability bias, which could lead participants to underreport or over report their behaviors. Additionally, this study was unable to include participants from private health institutions.

### 4.2 Conclusion and recommendation

This study found a high prevalence of hazardous alcohol use among health professionals in Bahir Dar, Northwest Ethiopia, with key risk factors including psychological distress, family history of alcohol use, lower educational status, and khat use. These findings highlight the need for targeted interventions addressing mental health, familial influences, and substance use. The burden aligns with global research, reflecting common occupational stressors in healthcare. Psychological distress was a significant predictor of hazardous drinking, emphasizing the need for stress management and mental health support for healthcare workers. To reduce hazardous alcohol use, the study recommends mental health programs, workplace policies promoting well-being, and integrated prevention and treatment strategies for alcohol and khat use. These efforts can improve healthcare worker mental health, work-life balance, and overall health outcomes.

## Recommendation

Establish Mental Health Programs for Health Professionals: Mental health programs should be created to address stress management, emotional well-being, and healthy coping strategies, with trained mental health professionals available to support healthcare workers facing burnout or distress.

Develop and Train Counselors for Healthcare Workers: Counselors specializing in healthcare worker stress should be trained to offer tailored support, including managing alcohol use disorders and providing both preventive and therapeutic interventions.

Conduct Regular Well-Being Assessments and Monitoring: Routine mental health evaluations and screenings for hazardous alcohol use (e.g., using AUDIT) should be conducted to identify early signs of distress and intervene proactively.

Implement Workplace Policies to Address Alcohol Use: Workplace policies should regulate alcohol use at professional events to reduce social drinking pressures and encourage responsible consumption.

Create Confidential Reporting Systems and Access to Rehabilitation Programs: Confidential reporting systems for alcohol-related issues should be established, along with accessible rehabilitation programs, ensuring health professionals can seek help without fear of stigma.

Promote Work-Life Balance and Regulated Working Hours: Policies promoting work-life balance, regulated hours, and adequate leave are essential to reduce burnout and prevent reliance on alcohol as a coping mechanism.

Develop Integrated Prevention and Treatment Programs Focused on Alcohol and Khat Use: Integrated programs addressing both alcohol and khat use should be implemented, offering combined behavioral therapies for health professionals.

## Data Availability

The original contributions presented in the study are included in the article/supplementary material. Further inquiries can be directed to the corresponding author.
