# Supplementary material for: Exploring hazardous alcohol use and its determinants among health professionals in Bahir Dar, Northwest Ethiopia
Source: Front Psychiatry. 2025 Feb 20;16:1511575. doi: 10.3389/fpsyt.2025.1511575 (PMC11883138; doi:10.3389/fpsyt.2025.1511575)
Supplement: Supplementary file 1 [file Table1.docx]

English version Questionnaires

**Instruction**:

The Questionnaires has six sections in order to assess the prevalence and associated factors of hazardous alcohol use among Health professionals in Bahir Dar city, North West Ethiopia. Give your answer by encircle the best alternative or put ‘X’ mark. Additionally, fill your answer on the provided space in the table. It will take 15-20 minutes to complete. Please try to respond all questions. Thank you very much for your patience.

**SECTION 1: Socio demographic characteristics**

| **No** | **Question** | **Answer** | **Code** |
| --- | --- | --- | --- |
| 101 | How old are you? |  |  |
| 102 | Sex | Male  Female |  |
| 103 | What is your marital status? | 1.Single  2.married  3.Divorced  4.Widowed  5. separated |  |
| 104 | What is your religion? | 1.Orthodox Christian  2. Muslim  3. protestant  4. Others |  |
| 105 | What is your ethnicity? | Amhara  Oromo  Tigry  Guragie  Other_________ |  |
| 106 | What is your profession? |  |  |
| 107 | What is your educational level? | Diploma  Fist degree  Second degree  PHD  Post-doctoral |  |
| 108 | What is your monthly income? | ----------------------------- |  |
| 109 | What is your Work experience? |  |  |
| 110 | With whom you are living currently? | Family  Alone  Relative  Friends  Others specify_____ |  |
| 111 | Does anyone in your family members drink alcohol? | **Yes**  **No** |  |
| 112 | Does anyone in your friends drink alcohol? | **Yes**  **No** |  |

**Section two: Alcohol use disorder identification test questionnaire (AUDIT)**

| **No** | **Questions** | | **Choice of Answers** | **Skip to code** | **Score** |
| --- | --- | --- | --- | --- | --- |
| Q 201 | How often do you have a drink containing alcohol? | | 1.Never  2.Monthly or less  3.2 to 4 times a month  4. 2 to 3 times a week  5. 4 or more times a week | If your answer is never for Q 201 go to Q 301 |  |
| 202 | A | What type of alcohol do you drink? | 1 Tella  2. Areki  3. Teji  4. woine  5. Birr  6. other____________ |  |  |
|  | B | How many standard drinks with respective drinking receptacles (1 standard drinking equivalent to 1 Tasa /thin Tella, 1 Brille Teji, 1 Melekia Arake, 1 bottle beer, 1 Single Draft, 1 glass Wine) do you have on a typical day when you are drinking? | 1 or 2  3 or 4  4 or 5  7 or 9  10 or more |  |  |
| **203** | How often do you have 6 or more standard drinks on one occasion? | | 1.Never  2.Less than monthly  3.Monthly  4.Weekly  5.Daily or almost daily |  |  |
| **204** | How often during the last year have you found that you were not able to stop drinking once you had started? | | 1. Never  2. Less than monthly  3. Monthly  4.Weekly  5. Daily or almost daily |  |  |
| **205** | How often during the last year have you failed to do what was normally expected from you because of drinking? | | 1. Never  2. Less than monthly  3. Monthly  4. Weekly  5. Daily or almost daily |  |  |
| **206** | How often during the last year have you needed a drink in the morning to get yourself going after a heavy drinking session the previous night? | | 1. Never  2. Less than monthly  3. Monthly  4. Weekly  5. Daily or almost daily |  |  |
| **207** | How often during the last year have you had a feeling of guilt or remorse after drinking? | | 1. Never  2. Less than monthly  3. Monthly  4. Weekly  5. Daily or almost daily |  |  |
| **208** | How often during the last year have you been unable to remember what happened the night before because you had been drinking? | | 1. Never  2.Less than monthly  3. Monthly  4. Weekly  5. Daily or almost daily |  |  |
| **209** | Have you or someone else been injured as a result of your drinking? | | 0. No  2.Yes, but not in the last year  4.Yes, during the last year |  |  |
| **210** | Has a relative or friend or a doctor or another health worker been concerned about your drinking or suggested you cut down? | | 0. No  2. Yes, but not in the last year  4.Yes, during the last year |  |  |
| **211** | What are your reasons for drinking alcohol? (more than one answer is possible) | | Low interest in profession  Poor relationship with managers,  Job insecurity  To forget stress (for relaxation)  Worry of getting infection like , HIV AIDIS, Hepatitis, COVID-19/others  Performance enhancement (long working hours)  Peer pressure  Family problems and low self-esteem |  |  |
| **212** | Do have contact history with person with COVID-19 in the past 14 days? | | Yes  No |  |  |
| **213** | Do you fear being infected with COVID-19 at work? | | Yes  No |  |  |

**Section Three: Kessler Test**

**Instruction:** These questions concern how you have been feeling over the past 30 days. Tick a box below each question that best represents how you have been.

| **No** | **Question** | **None of the time** | **A little of the time** | **Some of the time** | **Most of the time** | **All of the time** |
| --- | --- | --- | --- | --- | --- | --- |
| **301** | During the last 30 days, about how often did you feel tired out for no good reason? |  |  |  |  |  |
| **302** | During the last 30 days, about how often did you feel nervous? |  |  |  |  |  |
| **303** | During the last 30 days, about how often did you feel so nervous that nothing could calm you down? |  |  |  |  |  |
| **304** | During the last 30 days, about how often did you feel hopeless? |  |  |  |  |  |
| **305** | During the last 30 days, about how often did you feel restless or fidgety? |  |  |  |  |  |
| **306** | During the last 30 days, about how often did you feel so restless you could not sit still? |  |  |  |  |  |
| **307** | During the last 30 days, about how often did you feel depressed? |  |  |  |  |  |
| **308** | During the last 30 days, about how often did you feel that everything was an effort? |  |  |  |  |  |
| **309** | During the last 30 days, about how often did you feel so sad that nothing could cheer you up? |  |  |  |  |  |
| **310** | During the last 30 days, about how often did you feel worthless? |  |  |  |  |  |

**Section Four: Using Substances Other Than Alcohol**

**Instruction:** The following question asks you about your experience of using these substances across your lifetime and in the past three months. These substances can be smoked, swallowed, sniffed, injected or taken in the form of pills. Please be assured that information on such use was treated.

|  | **Questioners** | **Alternative response** | **Code** |
| --- | --- | --- | --- |
|  | **In your life have you ever used the following substances? (NON-MEDICAL USE ONLY)?** |  |  |
| 401 | Tobacco products (cigarettes, chewing tobacco, cigars, etc) | 1,Yes  2.No |  |
| 402 | Amphetamine type stimulants (chat)? | 1,Yes  2.No |  |
| 403 | pethedine? | 1,Yes  2.No |  |
|  | **In the past 03 months, which of the following substances have you ever used? (NON-MEDICAL USE ONLY)?** |  |  |
| 404 | Tobacco products (cigarettes, chewing tobacco, cigars, etc) | 1,Yes  2.No |  |
| 405 | Amphetamine type stimulants (chat)? | 1,Yes  2.No |  |
| 406 | Pethedine? | 1,Yes  2.No |  |

**Section Five; Social Support**

**Instruction:** this part of the questionnaire contains 3 questions regarding your experience of social support and related issues. Please put (√) Mark On The response that is applicable to you.

|  |  | **Response** | | | | |
| --- | --- | --- | --- | --- | --- | --- |
| **S. No** | **Item** | **1** | **2** | **3** | **4** | **5** |
| 501 | How many people are so close to you that you can count on them if you have serious personal problems (choose one option)? | None | 1 or2 | 3-5 | More than 5 |  |
| 502 | How much concern do people show in what you are doing (choose one option)? | No concern and interest | Little concern and interest | Uncertain | Some concern and interest | a lot of concern and interest |
| 503 | How easy is it to get practical help from neighbors if need it? you should | Very difficult | Difficult | Possible | Easy | Very easy |

**Section Six**: **Job Characteristics and Level of Workplace Stress among Health Professionals**

| **No** | **Part I: job characteristics** | **Alternatives** |
| --- | --- | --- |
| **601** | How much time you spend on work per a week? |  |
| **602** | How much time you spend on working in night shift per a week? |  |
| **603** | How much time you spend working on weekends? |  |

| **No** | **Part II: work related stress** | **Never** | **Almost never** | **Sometimes** | **Fairly often** | **Very often** |
| --- | --- | --- | --- | --- | --- | --- |
| **613** | In the last month, how often have you been upset because of something happened unexpectedly at work? | 0 | 1 | 2 | 3 | 4 |
| **614** | In the last month, how often have you felt that you were unable to control the important things in your life? | 0 | 1 | 2 | 3 | 4 |
| **615** | In the last month, how often have you stressed at work? | 0 | 1 | 2 | 3 | 4 |
| **616** | In the last month, how often have you felt confident about your ability to handle work related problems? | 4 | 3 | 2 | 1 | 0 |
| **617** | In the last month, how often have you felt that things were going your way at work? | 4 | 3 | 2 | 1 | 0 |
| **618** | In the last month, how often have you found that you could not cope with all the demands of your job? | 0 | 1 | 2 | 3 | 4 |
| **619** | In the last month, how often have you been able to control irritation at work? | 4 | 3 | 2 | 1 | 0 |
| **620** | In the last month, how often have you felt that you were on top of work related tasks? | 4 | 3 | 2 | 1 | 0 |
| **621** | In the last month, how often have you been angered because of things that happened were outside of your control at work? | 0 | 1 | 2 | 3 | 4 |
| **622** | In the last month, how often have you felt job tasks were piling up so high that you could not complete them? | 0 | 1 | 2 | 3 | 4 |
